# Supplementary material for: Cobalt Phthalocyanine-Ionic Liquid Composite Modified Electrodes for the Voltammetric Detection of DNA Hybridization Related to Hepatitis B Virus
Source: Micromachines (Basel). 2021 Jun 26;12(7):753. doi: 10.3390/mi12070753 (PMC8306960; doi:10.3390/mi12070753)
Supplement: Supplementary file 1 [file micromachines-12-00753-s001.zip › micromachines-1227150-supplementary.pdf]

Supplementary Materials

Cobalt phthalocyanine-ionic liquid composite modified electrodes for voltammetric detection of DNA hybridization related to Hepatitis B virus

The stock solution of fish sperm double-stranded DNA (fsDNA, Sigma, Germany) was prepared as 1000 µg/mL in Tris-ethylenediaminetetraacetic acid (EDTA; TE) buffer solution (10 mM Tris-HCl, 1 mM EDTA, pH 8.00) that kept frozen. The diluted solutions of fsDNA were prepared by using 0.50 M acetate buffer (ABS) containing 20 mM NaCl (pH 4.80). The DNA probes and complementary DNA oligonucleotides were purchased (as lyophilized powder) from TIB Molbiol (Germany). DNA oligonucleotides linked from 5' end with different groups (-PO_4, -NH_2) were used in the optimization studies.

The base sequences of DNA oligonucleotides used in our study were given as below:

DNA oligonucleotides:

5'-$(CH_2)_6$-AGG GTG TCT GAA GGA GGG GG-3'

5'-NH_2-$(CH_2)_6$-AGG GTG TCT GAA GGA GGG GG-3'

5'-PO_4-$(CH_2)_6$-AGG GTG TCT GAA GGA GGG GG-3'

HBV DNA probe:

5'-NH_2-$(CH_2)_6$- AAT ACC ACA TCA TCC ATA TA-3'

Target (complementary of HBV DNA probe):

5'-TAT ATG GAT GAT GTG GTA TT-3'

Non-complementary (NC):

5'-AAT ACC TGT ATT CCT CGC CTG TC-3'

Single base mismatch (MM):

5'-TAT ATG GAT GAT GTG TTA TT-3'

The stock solutions of oligonucleotides (500 µg/mL) were prepared with Tris-EDTA buffer (10 mM Tris–HCl, 1 mM EDTA, pH:8.00; TE), which then kept frozen. More diluted solutions of DNA oligonucleotides, probe, HBV target, NC or MM sequences were prepared in 0.5 M phosphate buffer solution containing 20 mM NaCl (pH: 7,40; PBS).

Preparation of CoPc-IL solution

50 μg/mL of CoPc was firstly dispersed in dimethyl formamide (DMF) and this solution was kept in a sonicator for 90 min. The amount of 5% IL was then added into CoPc solution and this mixture was sonicated for 30 min.

Preparation of CoPc-IL-PGEs

PGEs were pretreated by applying +1.40 V for 30 s in acetate buffer solution (pH 4.80, ABS). Each pretreated pencil lead was immersed into the eppendorf tubes containing 100 μL of CoPc-IL solution and kept during 15 min. Each of CoPc-IL modified electrodes was dried at upside-down position during 30 min.

DNA immobilization on CoPc-IL-PGE

CoPc-IL-PGEs were immersed in 100 μL of 2.5 μg/mL fsDNA solution for 30 min. Then, these electrodes were washed with PBS for 10 s to remove unbound DNA from the surface of electrode.

Hybridization of HBV probe and HBV target or other oligonucleotides; NC, MM

HBV DNA probe, HBV target, NC and MM were prepared in PBS (pH 7.40). For the preparation of mixture sample containing a required amount of DNA probe and its target, they were mixed during 5 min.

The hybridization between 0.5 μg/mL amino linked DNA probe and 12.5 μg/mL HBV target, or other oligonucleotides; such as, NC or MM instead of target sequence was also performed in PBS.

Immobilization of hybrids of DNA probe and its target onto the surface of CoPc-IL-PGEs

CoPc-IL-PGEs were immersed into the vials containing 40 μL of sample of DNA-DNA hybrids during 30 min. Electrodes were then rinsed with PBS (pH 7.40) for 10 s.

Electrochemical Measurements

CV measurements were applied by scanning from −0.5 to +1.30 V and the scan rate as 50 mV/s. All CV measurements were executed in 2 mM $Fe(CN)_6^{3-/4-}$ containing 0.1 M KCl.

DPV measurements were studied by scanning from 0.00 V to +1.45 V at 50 mV the pulse amplitude with the scan rate 50 mV/s for measuring the oxidation signal of guanine. All DPV measurements were executed in acetate buffer solution (ABS) (pH 4.80).

All of the raw data was processed using the Savitzky and Golay filter (level 2) of the NOVA software.

EIS measurements were executed in 2.5 mM $Fe(CN)_6^{3-/4-}$ containing 0.1 M KCl. The impedance was measured in the frequency range between 100 mHz and 100 kHz at a potential of +0.23 V. Sinusoidal signal was 10 mV. The respective semicircle diameter corresponds to the charge-transfer resistance, R_{ct}, the values of which are calculated using the fitting

programme AUTOLAB PGSTAT30 NOVA 1.11 software. The frequency interval divided into 98 logarithmically equidistant measure points.

Microscopic Characterization of The Electrodes

The microscopic characterization of PGE, CoPc-PGE, IL-PGE and CoPc-IL-PGE was performed using SEM (Quanta 400 FEI, Tokyo, Japan) with acceleration voltage 10 kV with the resolution in various magnitudes; 2 μm and 10 μm.

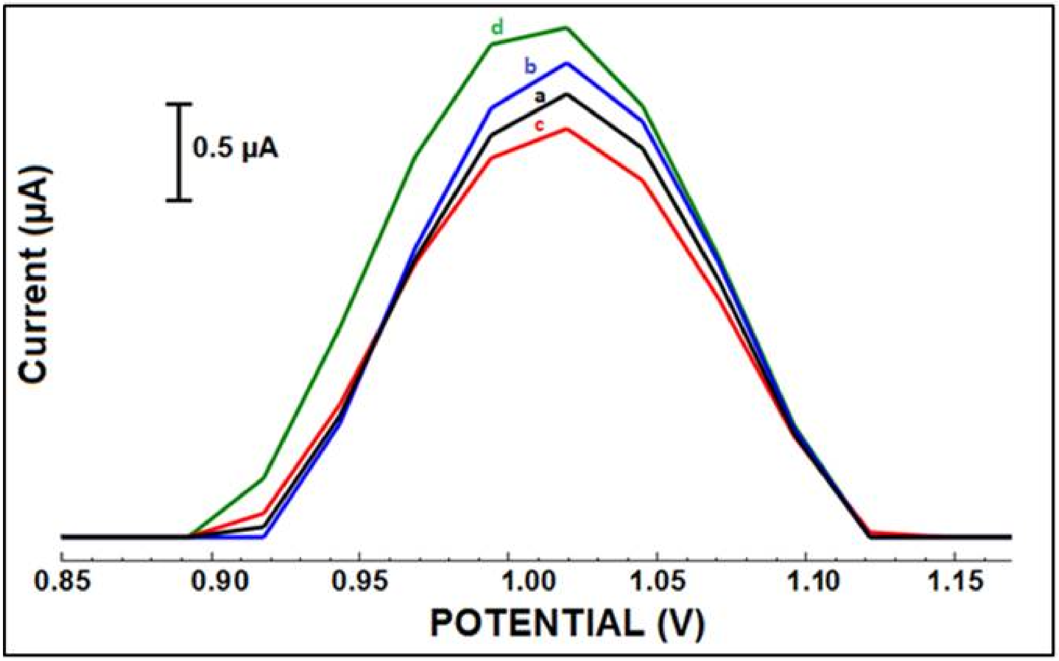

Figure S1. DPVs representing the guanine oxidation signal measured by using 2.5 μg/mL fsDNA immobilized onto the surface of (a) PGE, (b) IL-PGE, (c) CoPc-PGE, (d) CoPc-IL-PGE.

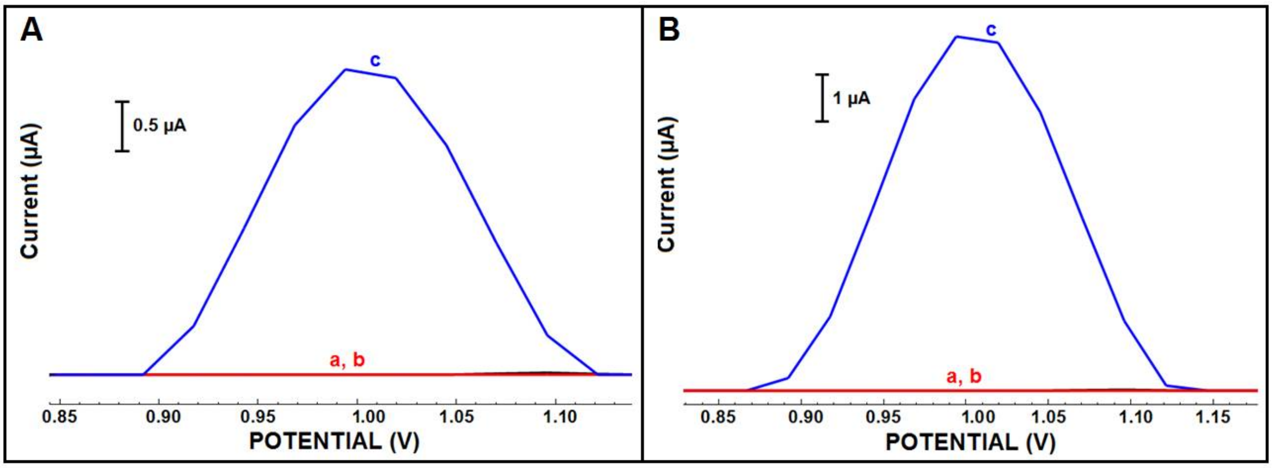

Figure S2. DPVs representing the signals measured at (a) control experiment of CoPc-IL-PGE in ABS, and (b) the guanine oxidation signal of amino linked HBV probe immobilized CoPc-IL-PGE before hybridization, (c) the guanine oxidation signal after hybridization occurred between amino linked HBV probe and HBV target by following the hybridization process: (A) step by step and (B) solution phase.

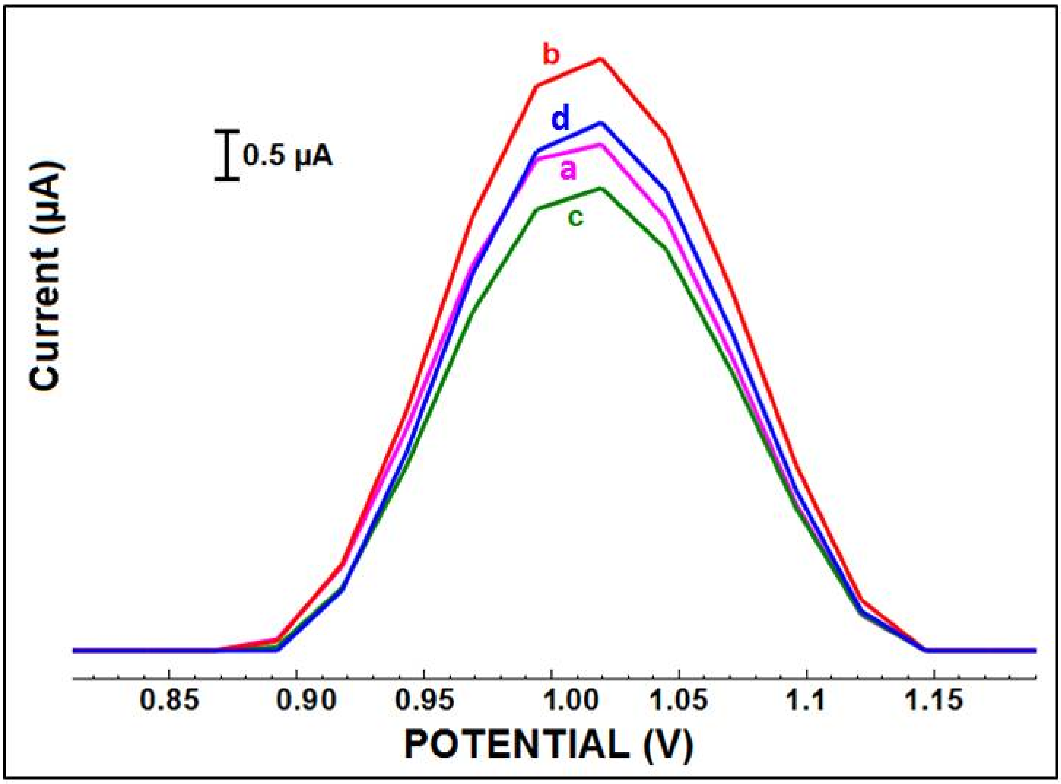

Figure S3. DPVs representing the guanine oxidation signals observed after hybridization between (a) 0.25, (b) 0.5, (c) 0.75, (d) 1 µg/mL amino linked DNA probe and 5 µg/mL HBV target.

Table S1. The average guanine oxidation signal (n=3) measured in the presence of hybridization of DNA probe with 5 µg/mL HBV target in various concentrations of DNA probe.

Probe Concentration (µg/mL)	Guanine oxidation signal (µA)	RSD %
0.25	6.00 ± 0.86	14.28
0.50	6.29 ± 0.13	2.13
0.75	5.35 ± 1.14	21.40
1	6.17 ± 1.01	16.42

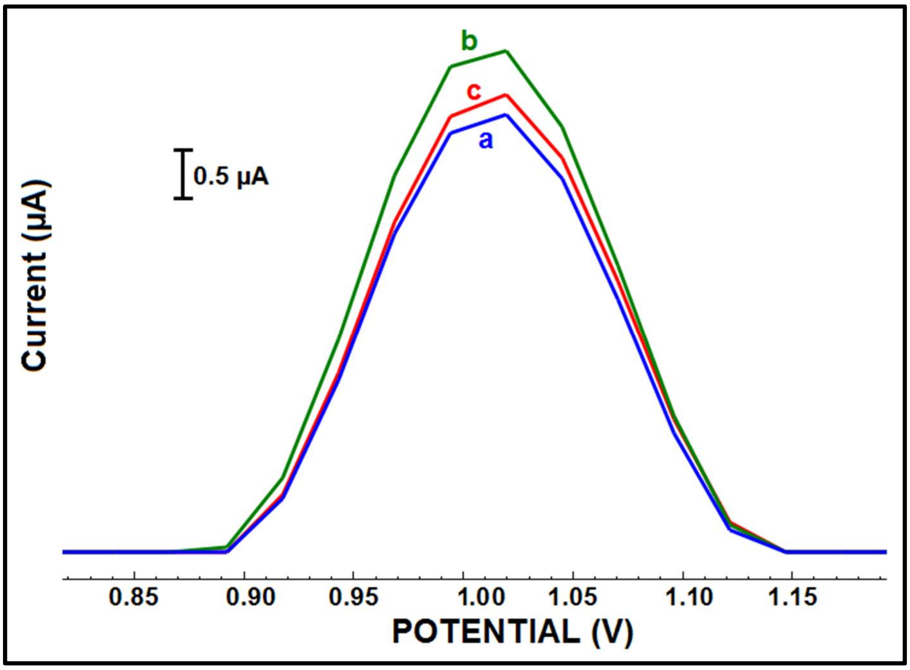

Figure S4. DPVs represented the guanine oxidation signals after hybridization between 0.5 µg/mL amino linked HBV probe and 5 µg/mL HBV target during (a) 3 min, (b) 5 min, (c) 15 min hybridization time.

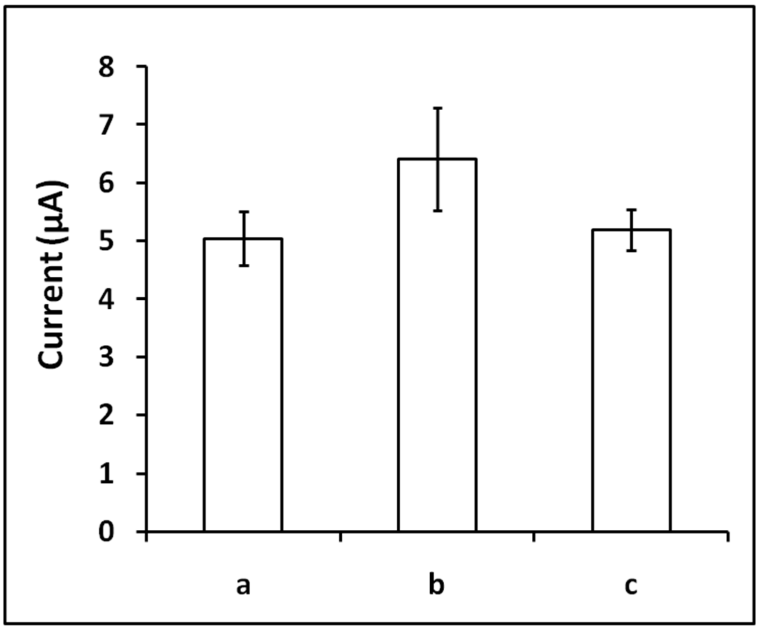

Figure S5. Histograms represented the average guanine signals (n=3) after hybridization between 0.5 μg/mL amino linked HBV probe and 5 μg/mL HBV target during (a) 3 min, (b) 5 min, (c) 15 min hybridization time.

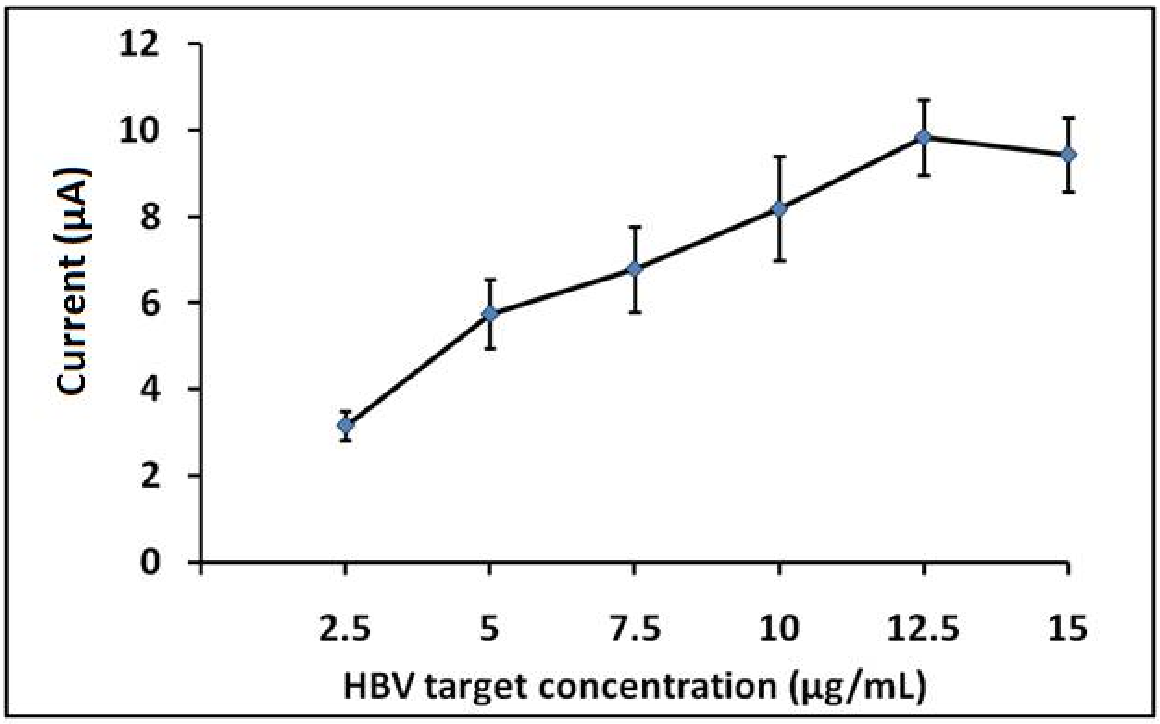

Figure S6. Line graph obtained from the average guanine signals (n=3) after hybridization of amino linked HBV probe with HBV target in its various concentrations.

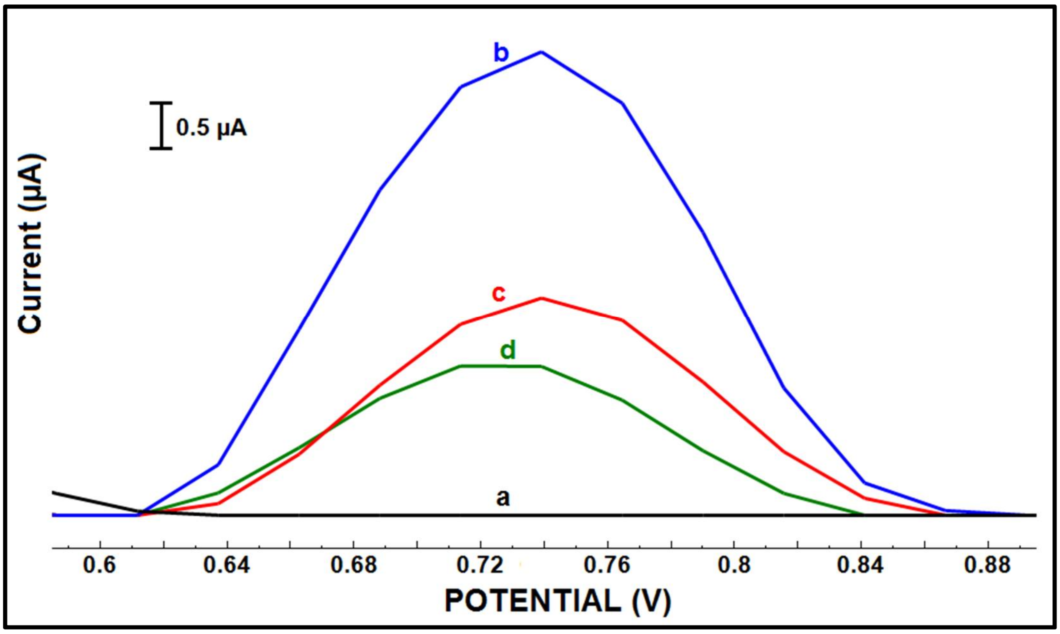

Figure S7. DPVs representing (a) control experiment of CoPc-IL-PGE in ABS, the oxidation signals of (b) FBS, (c) 1:100, (d) 1:500 FBS:PBS diluted solution.

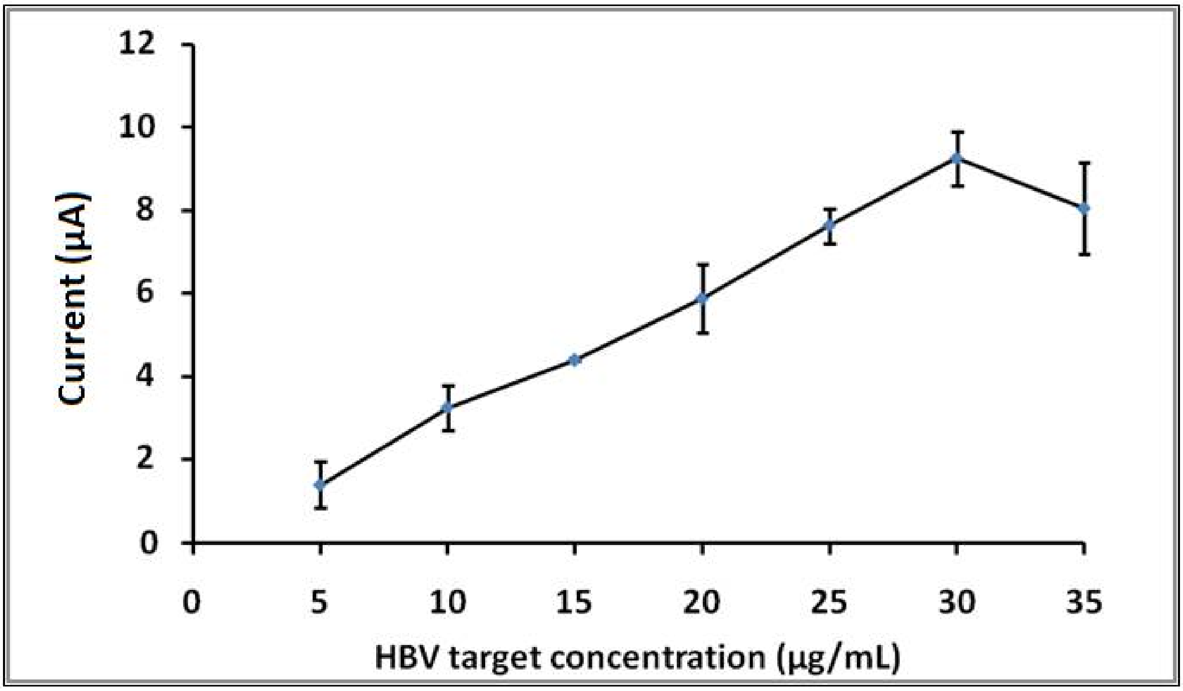

Figure S8. Line graph obtained from the average guanine signals (n=3) after hybridization of amino linked HBV probe with HBV target in its various concentrations in the medium of FBS.

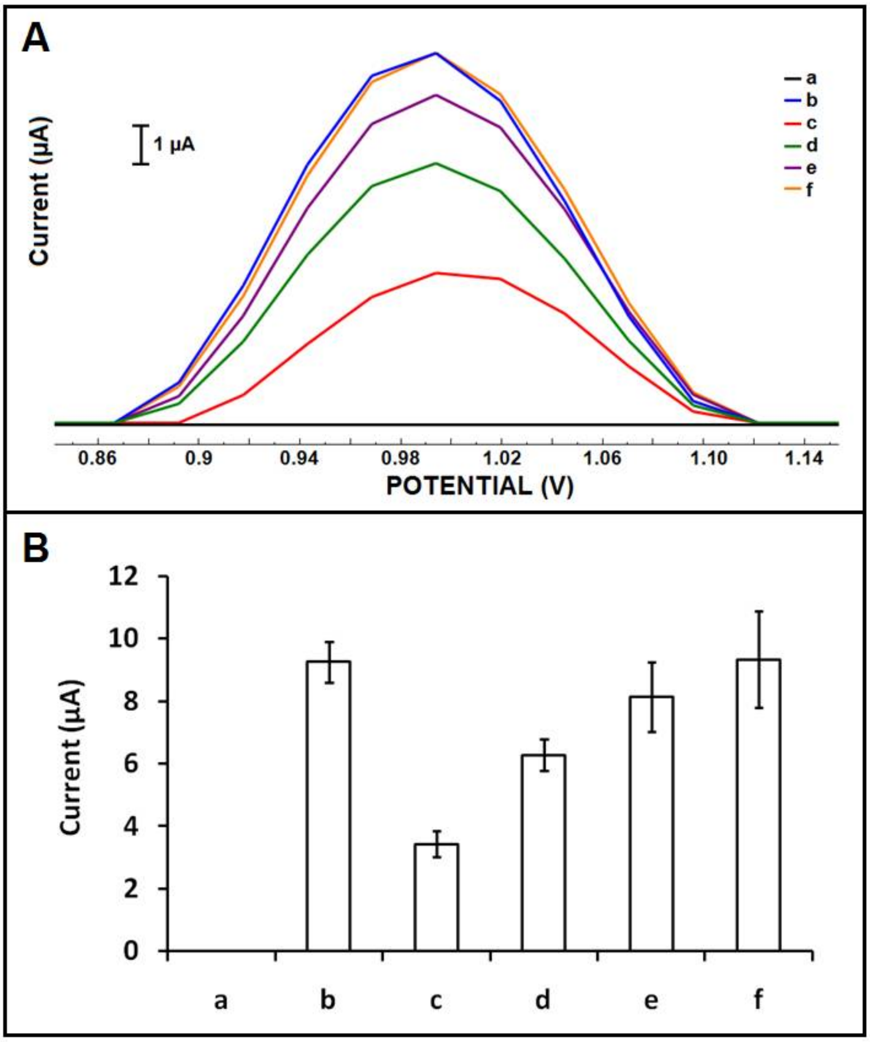

Figure S9. (A) Voltammograms and (B) histograms representing the average guanine oxidation signal obtained by (a) 0.5 µg/mL HBV probe immobilized CoPc-IL-PGE, after hybridization of amino linked HBV probe with 30 µg/mL (b) HBV target in FBS medium, (c) NC, (d) MM, the mixture of (e) HBV target: NC (1:1), (f) HBV target: MM (1:1) in FBS medium (n=3).
